# PEDF and PEDF-derived peptide 44mer stimulate cardiac triglyceride degradation via ATGL

**DOI:** 10.1186/s12967-015-0432-1

**Published:** 2015-02-21

**Authors:** Hao Zhang, Teng Sun, Xia Jiang, Hongli Yu, Meng Wang, Tengteng Wei, Huazhu Cui, Wei Zhuang, Zhiwei Liu, Zhongming Zhang, Hongyan Dong

**Affiliations:** Department of Thoracic and Cardiovascular Surgery, The First Affiliated Hospital of Nanjing Medical University, Nanjing, 210029 China; Department of Thoracic Cardiovascular Surgery, Affiliated Hospital of Xuzhou Medical College, Xuzhou, 221006 China; Research Facility Center for Morphology, Xuzhou Medical College, Xuzhou, 221004 China

**Keywords:** Pigment epithelium-derived factor, 44mer, ATGL, Myocardial infarction, Triglyceride

## Abstract

**Background:**

Pigment epithelium-derived factor (PEDF) is a 50-kDa secreted glycoprotein that is highly expressed in cardiomyocytes. A variety of peptides derived from PEDF exerts diverse physiological activities including anti-angiogenesis, antivasopermeability, and neurotrophic activities. Recent studies demonstrated that segmental functional peptides of PEDF, 44mer peptide (Val78–Thr121), show similar neurotrophic and cytoprotective effect to that of the holoprotein. We found that PEDF can reduce infarct size and protect cardiac function after acute myocardial infarction (AMI). However, the effects of PEDF on cardiac triglyceride (TG) accumulation after AMI remain unknown. The present study was performed to demonstrate the influence of PEDF and its functional peptides 44mer on TG degradation in AMI.

**Methods:**

The left ascending coronary artery (LAD) was ligated to induce AMI. PEDF-small interfering RNA (siRNA)-lentivirus (PEDF-RNAi-LV) or PEDF-LV was delivered to the ischemic myocardium in order to knock down or overexpress PEDF, respectively. Oil Red O staining and a TG assay kit were used to analyze the TG content in cardiomyocytes and infarcted areas.

**Results:**

The TG content significantly decreased in the PEDF-overexpressing heart compared to the sham group (*P* < 0.05). Both rPEDF and 44mer administration stimulate the TG degradation in cultured cardiomyocytes (*P* < 0.05). Adipose triglyceride lipase (ATGL)-specific inhibitor, atglistatin, attenuated the PEDF or 44mer-induced TG lipolysis activation of cardiomyocytes at 10 μmol/L. The effects of PEDF and 44mer on myocardial TG degradation were also abolished when ATGL was downregulated.

**Conclusions:**

We conclude that PEDF and 44mer promote TG degradation in cardiomyocytes after AMI via ATGL. The substitution of PEDF and 44mer may be a novel therapeutic strategy for cardiac TG accumulation after AMI.

## Background

Acute myocardial infarction (AMI) is among the leading causes of death in western societies. During AMI, reduced oxygen availability promotes triglyceride (TG) accumulation in cardiomyocytes [1]. Fat accumulation is toxic to cardiomyocytes and plays an important role in the progression of cardiomyopathy and heart failure [2]. Insulin-sensitizing drugs can reduce the deposition of lipids in the myocardium and reverse contractile dysfunction in lipotoxic rats, suggesting that intramyocardial TG accumulation is deleterious [3]. Thus, reducing TG accumulation in cardiomyo- cytes in the ischemic heart is beneficial.

Pigment epithelium-derived factor (PEDF) is a member of the superfamily of serine protease inhibitors and was first identified in cultured retinal pigment epithelial cells [4]. It is expressed in many cell types, including adipocytes, hepatic cells and the cells of the eye. It is a multifunctional, pleiotropic protein with antiangiogenic, antioxidant, antithrombotic, neurotrophic and neuroprotective properties [5,6]. Clinical studies have demonstrated that the level of plasma PEDF was elevated in an obese population [7], as well as in metabolic syndrome [8] and type 2 diabetes patients [9]. Recent studies demonstrated that PEDF sourced from adipose tissue contributed to enhanced adipocyte lipolysis [10].

PEDF is thought to exert its biologic actions by binding to a cell surface receptor. A recent report noted that adipose triglyceride lipase (ATGL) is a receptor for PEDF in retinal epithelial cells and utilized a cell-free system to demonstrate that this interaction induced lipase activity [11]. ATGL is the key enzyme of TG catabolism and functions as a monoacyl hydrolase that catalyzes the initial, rate-limiting step of the TG lipolysis cascade [12]. The absence of this enzyme in cardiac muscle causes a lipolytic defect that result in massive lipid accumulation, severe cardiac dysfunction and premature death [13]. Petra C. Kienesberger found that the overexpression of ATGL in cardiomyocytes was sufficient to enhance myocardial TG hydrolysis, decrease the cardiomyocyte TG content, and influence cardiac function at baseline as well as during pathophysiological stress [14].

Recent studies demonstrated that a variety of peptides derived from PEDF exerts diverse physiological activities including anti-angiogenesis, vascular permeability resistance, and neuronal protective effect. Among these functional peptides, the 44mer peptide (aa78-121) derived from the N-terminal edge of PEDF had neurotrophic activity in retinoblastoma cells and cerebellar granuleneurons [15].

Our group found that PEDF can reduce the infarct size and protect cardiac function from ischemic injury. We also found that PEDF and its functional peptides 44mer protect cultured H9c2 cells and primary cardiomyocytes against apoptosis and necroptosis under hypoxic conditions [16]. However, no study to date has focused on the relationship between TG accumulation and PEDF or its derived peptide 44mer after AMI.

In the present study, we demonstrated the role of PEDF and its functional peptides 44mer in TG regulation and their underlying mechanism, which will provide novel information about the prevention and treatment of cardiac TG accumulation after AMI.

## Methods

### Recombinant lentivirus constructs and viral production

A recombinant lentivirus (LV) was prepared as described previously. PEDF over-expression plasmids and the RNAi vector PEDF-RNAi-LV of the PEDF gene that produces PEDF shRNA were successfully constructed and then successfully packaged by 293 T cells. The concentrated titer of the virus suspension was 2 × 10^12^ Tu/L.

### Reagents

Recombinant PEDF(rPEDF) was synthesized by CUSABIO BIOTECH CO. Ltd. Synthetic peptide 44mer was designed from amino acid positions 78–121 (ILLSPLSVATAL SALSLGAEQRTESVIHRALYYDLINNPDIHST) of the rat PEDF sequence (GenBankTM accession number NM_177927), and prepared by GL Biochem (Shanghai) Ltd. Atglistatin was purchased from MedKoo Biosciences (USA). Polyclonal goat anti-ATGL antibody and polyclonal rabbit anti-PEDF antibody from Santa Cruz were used in this study.

### Experimental animal

Neonatal Sprague–Dawley (SD) rats (1–3 d old, weighing 5–7 g) and Male Sprague Dawley rats, 200–250 g, were purchased from the Experimental Animal Center of Xuzhou Medical College. All animal experiments were conducted in accordance with the Guide for the Care and Use of Laboratory Animals published by the US National Institutes of Health (NIH Publication, 8th Edition, 2011). The animal care and experimental protocols were approved by the Xuzhou Medical College Committee on Animal Care.

The rats were anesthetized with intraperitoneal injections of sodium pentobarbital (60 mg/kg, i.p.), and maintained by bolus injections of sodium pentobarbital (3–6 mg/kg, i.v.) during anesthesia as required. A left thoracotomy through the fourth intercostal space was performed under sterile conditions on a volume-cycled ventilator SAR-830 (Bio Research Center Inc., Na-goya, Japan). The left-anterior descending coronary artery (LAD) was then ligated with a 6–0 silk suture (Ethicon, Johnson & Johnson, USA) 2 mm distal from its origin. Sham-operated rats underwent the same procedure, excluding coronary artery ligation.

For intramyocardial gene delivery, PEDF-LV or PEDF-RNAi-LV (2 × 10^7^ TU) in 20 μL Enhanced Infection Solution (ENIS, pH 7.4) was delivered with a 20-μL syringe and 25-gauge needle into three sites the myocardium along the infarct border immediately after surgery. Control animals received an equivalent volume of lentivirus vector expressing the green fluorescent protein (GFP) reporter genes. The validity of myocardial viral vector transfection was obtained as described previously [17].

### Primary cardiomyocyte isolation and cell culture

Ventricular myocytes were isolated from neonatal rats as previously described [16]. The harvested cells were plated on glass coverslips in 12-well plates (Corning, USA) at 1 × 10^5^ cells per well for oil red O and immunofluorescence staining or in 6-well plates (Corning, USA) for TG contend analysis. More than 90% of the cells were cardiomyocytes, as evaluated by indirect immunofluorescence staining with a monoclonal anti-actin (a-sarcomeric) antibody (A2172, Sigma). The cells were cultured in 5% CO_2_ at 37°C for 72 h prior to the hypoxia treatments. Hypoxic conditions were induced by placing cells in a hypoxia chamber (Heal Force, Shanghai, China) with a water-saturated atmosphere composed of 5% CO_2_/3% O_2_ for 48 h. All experiments were performed in accordance with relevant guidelines and regulations.

### Oil red O staining

The oil Red O solution was purchased from Sigma-Aldrich. The cells were washed twice with PBS and fixed with 4% formaldehyde in PBS for 5 minutes. After three washes in PBS, the cells were briefly washed with 60% isopropanol and incubated with 60% filtered oil Red O solution (0.7 g per 200 ml of isopropanol) for 30 min at room temperature. The dishes were then rinsed in 60% isopropanol. To determine cardiac lipid accumulation, frozen sections of heart (7 μm) were fixed with 4% formaldehyde in PBS, stained with oil Red O for 30 minutes and washed three times. Representative photomicrographs were captured at 200× magnification using a system incorporated in the microscope (Olympus, Japan).

### Triglyceride assay

The levels of intracellular and heart TGs were assessed using a TG assay kit (GPO-POD; Applygen Technologies Inc., Beijing, China) according to the manufacturer’s recommended protocol. Intracellular free fatty acids were estimated using an ultrasensitive assay kit for free acids (Applygen Technologies Inc.) according to the manufacturer’s recommended protocol.

### Western blot analysis

The cells were washed and scraped with PBS and incubated in an RIPA buffer containing a protease inhibitor cocktail (Roche Diagnostics, Mannheim, Germany). For the western immunoblot analysis, protein samples (20 μg/lane) from cardiomyocytes were separated on 10% SDS-PAGE gels and transferred to a nitrocellulose membrane (Millipore, USA). The membranes were blocked for 1 h at room temperature with 5% Block-Ace (DS Pharma Biomedical, Osaka, Japan) in detergent-supplemented Tris-buffered saline (TBS-T; 20 mm Tris, 150 mm NaCl, 0.05% Tween 20, pH 7.5). The membranes were then incubated with anti-PEDF, anti-β actin or anti-ATGL antibodies overnight at 4°C, washed in TBS-T (3 × 10 min), and then incubated with fluorescently labeled secondary antibody (Rockland, USA) for 1 h at room temperature. After washing, the protein bands were scanned by the Odyssey Infrared Imaging System (Li-Cor Biosciences, USA).

### Quantitative qPCR–based gene expression

The total RNA was extracted from tissues or cells using TRIzol reagent (Invitrogen, Carlsbad, CA) and treated with RNase-Free DNase. Five hundred nanograms of total RNA was used for reverse transcription and quantitative real-time PCR analysis (qPCR). Real-time quantitative PCR was performed on a Light Cycler 480II (Roche, Switzerland) using SYBR Green PCR Master Mix (Applied Biosystems). The results were normalized to the expression of the GAPDH gene. The sequences of the forward and reverse primer were 5′- CATGGCCTTCCGTGTTCCTA −3′ and 5′- GCGGCACGTCAGATCCA −3′ for the GAPDH gene, 5′- CCAACGCCACTCACATCTAC −3′ and 5′- AGCAGGCAGGGTCTTCAGTA −3′ for the ATGL gene, 5’- CCGTAGTG GAGGAGGATGAC −3′ and 5’- ATCGTAGCCGAAGTTGGAAAC −3′ for the PEDF gene, 5′- TGACAGGGTGGCTTCCTAC −3′ and 5′- CGCAC AGGTTCTTTCTTGTAA −3′ for the CGI-58 gene.

### Immunoprecipitation

The cells were lysed in ice-cold buffer [in mmol/L: Mops (pH 7.4) 50, sucrose 320, KCl 100, MgCl 20.5, and inhibitors of proteases and phosphatases (β-glycerophosphate 20, sodium pyrophosphate 20, NaF 50, 1 mmol/L each of EDTA, EGTA, phenylmethylsulfonyl fluoride, benzamidine, sodium orthovanadate, and p-nitrophenyl phosphate, and 5 μg/mL each of aprotinin, leupeptin, and pepstatin A)]. The cell lysates were pre-cleared with 30 μl of immobilized protein A/G-agarose (Pierce) for 30 min on a rotator at room temperature and then centrifuged at 14,000 g and 4°C for 30 min. Two hundred and fifty microliters of the pre-cleared supernatant was incubated overnight at 4°C with appropriate antibodies diluted in immunoprecipitation buffer [in mmol/L: Hepes 50 (pH 7.4), NaCl 150, glycerol 10% (vol/vol), ZnCl 21, MgCl 21.5, Triton X-100 1%, Nonidet P-40 0.5%, and the phosphatase and protease inhibitors mentioned above]. The mixture was then incubated with 50 μl of protein A-Sepharose CL-4B at 4°C for an additional 2 h. The samples were then washed three times with cold immunoprecipitation buffer and eluted by boiling for 5 min in 4 × Laemmli sample buffer. The samples were separated on 4-12% polyacrylamide NuPAGE gels (Invitrogen), transferred to nitrocellulose membranes, and probed with the specific indicated antibodies, followed by a corresponding horseradish peroxidase-conjugated secondary antibody.

### Double immunofluorescence staining

Double-labeling immunofluorescence was performed to detect the ATGL and PEDF localization in cardiomyocytes. Cardiomyocytes were cultured on coverslips in tissue culture dishes. After fixation in 4% paraformaldehyde for 20 min at room temperature, the cells were washed 3 times with PBS (Phosphate Buffered Saline) and blocked for 30 min. Mouse anti-rat antibodies raised against actin (a-sarcomeric) (A2172, Sigma) were used as a cardiomyocyte marker protein. Rabbit anti-rat antibodies against PEDF (Santa Cruz, CA, USA) and goat anti-rat antibodies against ATGL (Santa Cruz, CA, USA) were applied to cells in different dishes and incubated overnight at 4°C. After washing, the PEDF- and ATGL-stained cells were incubated with Alexa Fluor® 594 anti-rabbit antibodies (life technologies) and Alexa Fluor® 488 rabbit anti-goat antibodies (life technologies), respectively, for 1 h at room temperature. The nuclei were counterstained with DAPI, and the cover slips were mounted on slides using 50% glycerin. The stained samples were photographed and analyzed using the TCS SP8 STED 3X (Leica, Germany).

### Electron microscopy

For transmission electron microscopy (TEM) observation, small samples of heart tissue were fixed in 2.5% glutaraldehyde overnight and then incubated while protected from light in 1% osmium tetroxide for 2 hours. After washing in distilled water, the specimens were incubated in 2% uranyl acetate for 2 hours at room temperature and then dehydrated in graded ethanol concentrations. Finally, the samples were embedded in molds with fresh resin. Ultrathin sections were obtained with an EM UC7 (Leica, Germany), stained with lead citrate and examined with a Tecnai G2 T12 (FEI, USA).

### Immuno-electron microscope experiment

Double immunocytochemical staining was applied for the detection of colocalization of PEDF with ATGL at electron microscopic level. Immuno-electron microscope experiment was employed based on the method described previously [18].

### Statistical analysis

The results are expressed as the means ± standard error. Statistical analysis of the results was carried out using the Student’s *t*-test or one-way analysis of the variance (ANOVA) followed by the Duncan’s new multiple range method or Newman-Keuls test. P < 0.05 was considered significant.

## Results

### Lipid droplets accumulated in ischemic rat heart tissue and hypoxic cardiomyocytes

**Figure 1:**
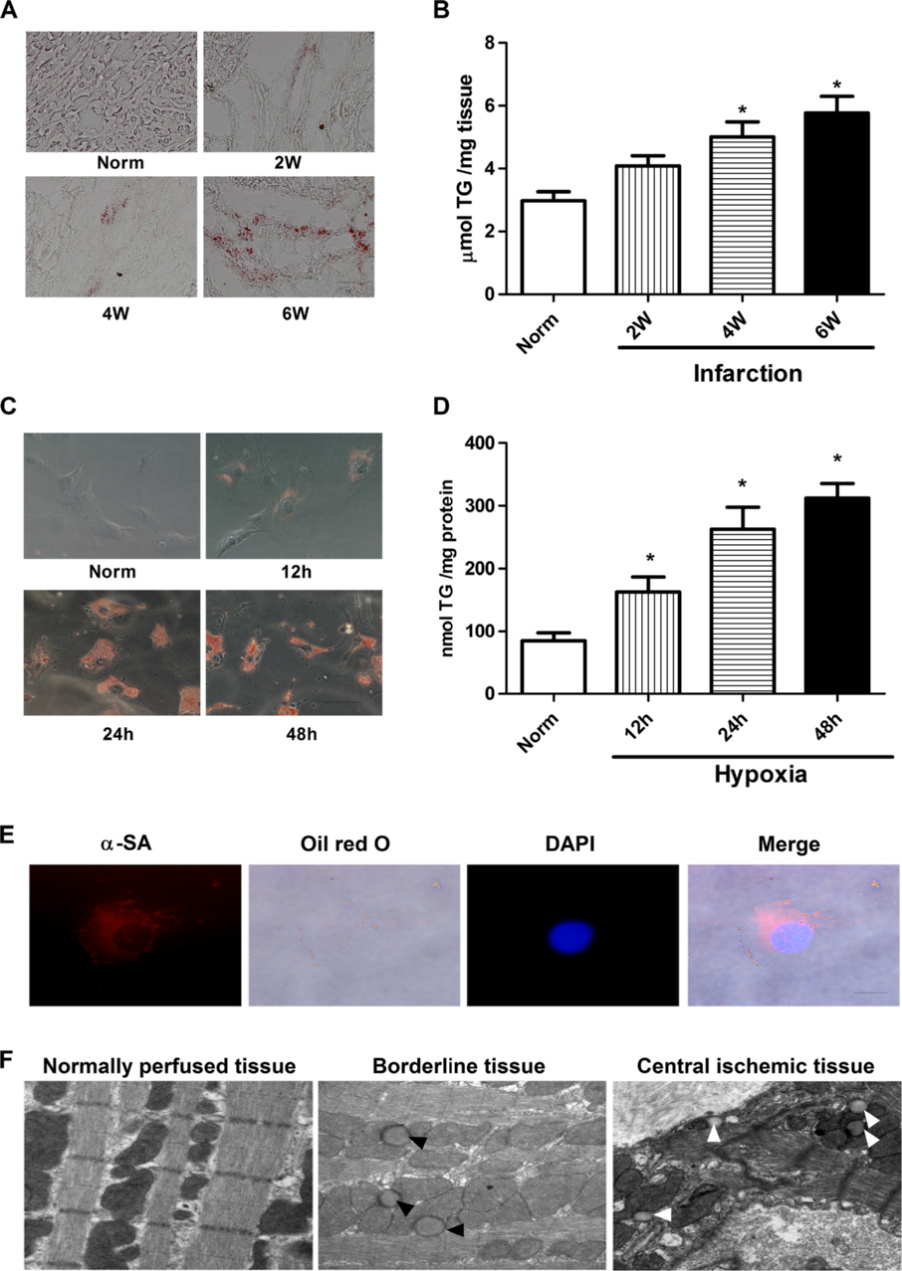
**Accumulation of neutral lipids in heart of infarcted rat and hypoxia cardiomyocytes. A**: lipid accumulation in the ventricular muscle from rat 2, 4, and 6 weeks after an experimentally induced myocardial infarction, as shown by oil red O staining (red). **B**: the TG content in hearts from rat 2, 4, and 6 weeks after an experimentally induced myocardial infarction. Values are means ± SD of at least 3 hearts. *P < 0.05 compared the normal group. Scale bar, 40 μm. **C**: lipid accumulation in cardiomyocytes incubated in normoxia condition or hypoxia conditions for 12, 24 and 48 hours. **D**: TG content in cardiomyocytes incubated in normoxia condition or hypoxia conditions for 12, 24 and 48 hours. *n* = 4, Values are means ± SD. *P < 0.05 compared with normal group. Scale bar, 20 μm. **E**: oil red O-positive cells were found to be α-sarcomeric actin positive myocytes. Nucleuses were stained with DAPI (blue). Scale bar, 10 μm. **F**: representative electron microscopic views of the normally perfused tissue, borderline tissue and central ischemic tissue from the left ventricular 4 weeks after myocardial infarct induction. Lipid droplets (arrowheads) are present in borderline and central ischemic tissue. All experiments were carried out three times. Scale bar, 500 nm.

Of the 20 rats entered into the present experiment, 4 died within 24 hours of surgery. Thus, 16 rats were available for morphometric and hemodynamic analyses and randomized into four groups (n = 5 at 2 weeks, n = 6 at 4 weeks, n = 5 at 6 weeks). As shown above (Figure 1A), substantial lipid accumulation was observed in the infarcted areas of the LV 2, 4 and 6 weeks after AMI, whereas no accumulation was visible in the normal group. A biochemical analysis of the muscle TG levels revealed a 1.6-fold increase in the cardiomyocyte TG content in infarcted rats 4 week after AMI compared with the normal controls (Figure 1B). Freshly isolated rat cardiomyocytes were incubated in 3% O_2_, which resulted in the accumulation of intracellular LD as evidenced by bright yellow dots in the micrographs of oil red O stained cells (Figure 1C). LDs could not be detected in normoxic cells. Furthermore, most of the cells were identified as cardiomyocytes because oil red O-positive cells were found to be cardiac myosin-positive myocytes (Figure 1E). Electron microscope images also showed a marked lipid accumulation in the borderline and ischaemic tissue 4 week after AMI compared with the normally perfused tissues (Figure 1F).

### Expression of PEDF and ATGL in the rat heart

**Figure 2:**
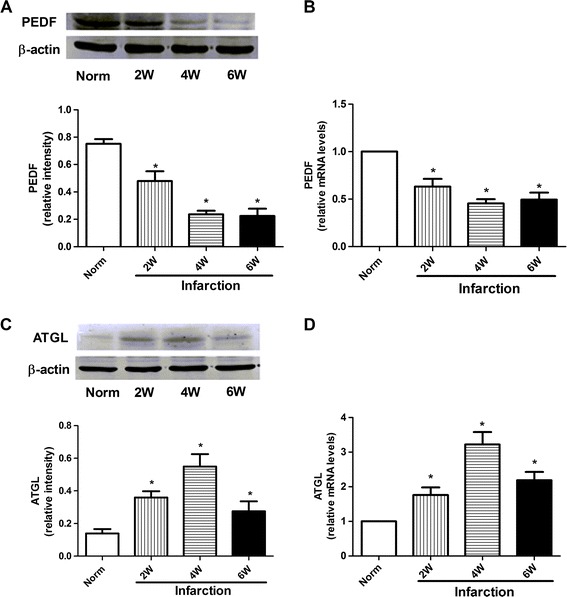
**Expression of PEDF and ATGL in the rat heart. A** and **C**: Expression of PEDF and ATGL protein 2, 4 and 6 weeks after infarction. Upper panels show representative results of PEDF and ATGL immunostaining. Lower panels show the quantitative data of PEDF and ATGL. β-actin was used as a loading control. The bands were quantified using the Image J software, and the data were transformed and normalized to β-actin. *n* = 3 per group. W indicates weeks. Values are means ± SD. *P < 0.05 compared with normal group. **B** and **D**: PEDF and ATGL mRNA expression in infarcted area 2, 4 and 6 weeks after infarction by qPCR analysis. NADPH was used as an internal control. *n* = 4, Values are means ± SD. *P < 0.05 compared with normal group.

The protein expression levels in LV after AMI at 2, 4 and 6 weeks were compared to the normal animals. As shown in Figure 2A, the protein levels of PEDF in the infarcted areas of LV significantly decreased 2 weeks after AMI compared with those of the normal group and remained low during the observational periods. However, the protein level of ATGL in infarcted areas of LV began to increase at 2 weeks and reached a maximum 4 weeks after LAD artery ligation; the peak value was 3.5-fold higher than the basal level (Figure 2C). Moreover, a similar decrease in PEDF transcripts and an increase in ATGL transcripts were observed in the qPCR assay in infarcted areas of LV at all time points. The level of PEDF decreased by 45% in infarcted hearts, whereas the ATGL levels increased 3-fold 4 weeks after infarction (Figure 2B and D). ATGL protein levels were significantly increased in the infarcted areas of LV compared to the normal group suggesting that cardiac TG accumulation is apparently controlled by the decrease of PEDF protein levels.

### PEDF and 44mer regulate TG degradation in cardiomyocytes

**Figure 3:**
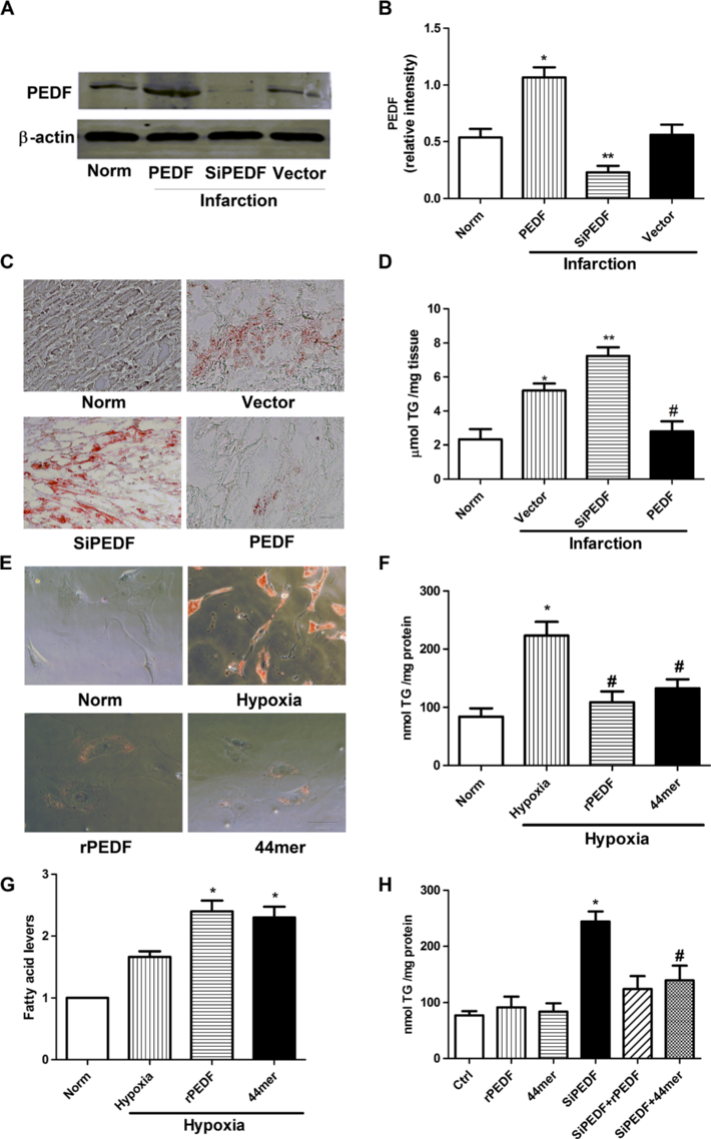
**PEDF and 44mer regulate TG degradation in cardiomyocytes. A**: western blot determination for PEDF protein expression in a rat AMI model. **B**: densitometry of the western blots shown in **(A)**, normalized to β-actin. *n* = 3, values are means ± SD. *P < 0.05 compared with normal group. **P < 0.05 compared with normal group and PEDF group. **C**: intracellular lipids were stained with oil Red O 4 weeks after AMI. The siPEDF group showed an abundance of lipid droplets, whereas the PEDF group showed very little staining. Scale bar, 40 μm. **D**: quantification of the lipid content in the heart on 4 weeks after AMI by biochemical methods. *n* = 3, values are means ± SD. *P < 0.05 compared with normal group; **P < 0.05 compared with vector group. #*P* < 0.05 compared with vector group and siPEDF group. **E**: the intracellular lipids were stained with oil Red O after 1 day of hypoxia. The hypoxia group showed an abundance of lipid droplets, whereas the rPEDF (10 nM) and 44mer (10 nM) groups showed very little staining. Scale bar, 20 μm. **F**: quantification of lipid content in cardiomyocytes after 1 day of hypoxia by biochemical methods. *n* = 3, values are means ± SD. *P < 0.05 compared with normal group; #*P* < 0.05 compared with hypoxia group. **G**: Influence of PEDF and 44mer on intracellular FFA under hypoxia condition. Values are means ± SD. *P < 0.05 compared with hypoxia group. **H**: PEDF downregulation in cardiomyocytes significantly increased the TG content compared to wild-type cardiomyocytes. The TG content did not significantly change in WT cardiomyocytes treated with rPEDF or 44mer, while the addition of rPEDF or 44mer to cardiomyocytes that expressed less PEDF significantly decreased the TG content. *n* = 3, values are means ± SD. *P < 0.05 compared with control group; #*P* < 0.05 compared with siPEDF group.

PEDF was down regulated in cardiomyocytes after AMI. Thus, to verify the potential function of PEDF in TG degradation, we delivered lentivirus carrying PEDF or PEDF RNAi by using intramyocardial injections to overexpress or knockdown PEDF in a rat AMI model (Figure 3A). In the infarcted zone, the myocardial TG content of the PEDF group was less than those of the other groups (Figure 3C and D). An examination of the siPEDF group revealed more fat accumulation in the cardiomyocytes than in the other groups. Cardiomyocytes incubated in 3% O_2_ for 24 hours were fixed with 4% paraformaldehyde, followed by staining with oil Red O.

In vitro, cardiomyocytes were incubated for 72 h under normal condition, and then treated with rPEDF (10nM) or 44mer (10nM) 6 h prior to the hypoxia treatments. We found the rPEDF and 44mer groups showed fewer and smaller lipid droplets in the cytoplasm than the hypoxia group (Figure 3E). Accordingly, the quantitation of TG in cardiomyocytes showed that the TG content of the rPEDF and 44mer groups were significantly lower than the hypoxia group (Figure 3F). Figure 3G showed a 20% and 18% increase in FFA levels with PEDF and 44mer as compared with hypoxia group (P < 0.05). We also performed the experiment in the absence of hypoxia stress. We found that the TG content is significantly lower under normal culture conditions in cardiomyocytes that expressed less PEDF than in wild-type cardiomyocytes. Treating wild-type cardiomyocytes with rPEDF or 44mer did not significantly affect the TG degradation in cultured cardiomyocytes, while PEDF downregulation significantly reduced the TG content in cardiomyocytes (Figure 3H). Taken together, our data suggest that PEDF and 44mer stimulate cardiac TG degradation.

### PEDF or 44mer on TG degradation was not due to increases in the expression of ATGL and CGI-58

**Figure 4:**
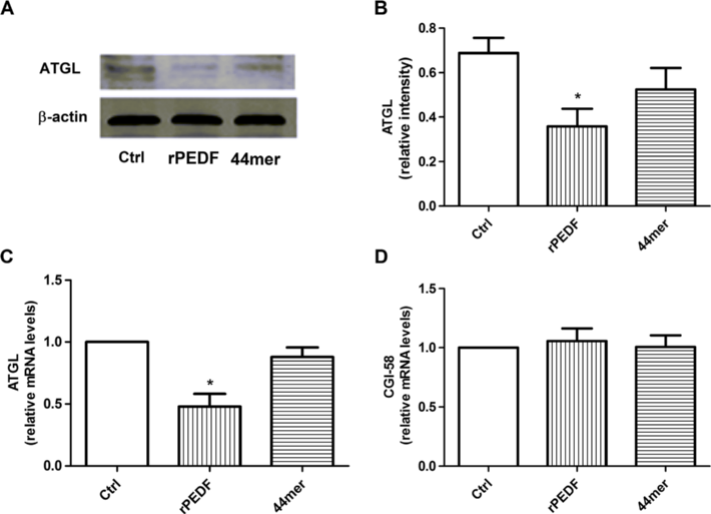
**Effects of PEDF and 44mer on ATGL and CGI-58 mRNA in cardiomyocytes. A**: western blot determination for ATGL protein expression, samples treated with rPEDF or 44mer. **B**: densitometry of the western blots shown in **(A)**, normalized to β-actin. n = 3, values are means ± SD. *P < 0.05 compared with control group. **C**: PEDF administration caused the ATGL mRNA levels to decrease. n = 5, values are means ± SD. *P < 0.05 compared with control group. **D**: PEDF and 44mer did not affect the expression of CGI-58 mRNA. n = 5, values are means ± SD.

ATGL was identified as a rate-limiting TG lipase, and its activity is stimulated by comparative gene identification-58 (CGI-58). Some studies have indicated that increased ATGL and CGI-58 expression could decrease the TG content. To determine whether the effect of PEDF or 44mer on TG degradation was due to increased ATGL and CGI-58 expression, the ATGL and CGI-58 mRNA levels were evaluated using qPCR. ATGL protein levels were not increased in the PEDF and 44mer groups compared to the control group (Figure 4A and B) suggesting that PEDF and 44mer function in the regulation of cardiac TG degradation was not apparently controlled by changes in ATGL protein expression levels. Furthermore, we used qPCR analysis to identify changes in the levels of ATGL mRNA. As shown in Figure 4C, rPEDF administration decreased the ATGL mRNA level while 44mer had no effect on the expression of ATGL. rPEDF or 44mer treatment did not change the level of CGI-58 mRNA compared to the control group (Figure 4D). These results indicated that the effect of PEDF or 44mer on TG degradation was not due to increases in the expression of ATGL and CGI-58.

### ATGL and PEDF co-localize to lipid droplets

**Figure 5:**
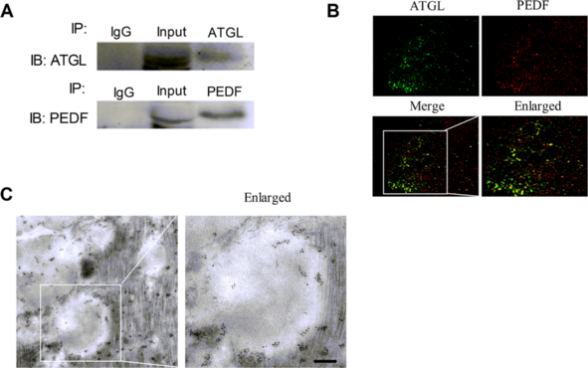
**ATGL and PEDF co-localize with lipid droplets in cardiomyocytes. A**: cardiomyocyte lysates were immunoprecipitated with ATGL or PEDF antibody, and the lysates were then detected with PEDF or ATGL antibodies. **B**: co-localization of PEDF (green) and ATGL (red) assessed via the double immunofluorescent labeling of cardiomyocytes. Yellow color indicates the merging of green and red. Scale bar, 2 μm. **C**: immunoelectron microscopy images showed that PEDF (labeled with gold particles of 5 nm in diameter) and ATGL (labeled with gold particles of 10 nm in diameter) co-localized around the lipid droplet and that the spatial distance between PEDF and ATGL was less than 20 nm. Scale bar, 100 nm.

A precipitate analysis indicated that full-length PEDF co-immunoprecipitated with full-length ATGL (Figure 5A) when anti-PEDF antibody was used, indicating a direct interaction between PEDF and ATGL. The co-immunoprecipitation experiment with anti-ATGL antibodies also co-immunoprecipitated ATGL and PEDF. To test whether ATGL and PEDF traffic to the lipid droplet in cardiomyocytes, the localization of PEDF and ATGL in rat primary cardiomyocytes was assessed using stimulated emission depletion (STED) microscopy imaging. As shown in Figure 5B, we found that PEDF co-localized with ATGL in the same discrete circular rings identified as lipid droplets by oil red O staining. An immuno-electron microscope experiment was also performed to evaluate the binding between endogenous PEDF and ATGL in cardiomyocytes. This experiment showed that PEDF and ATGL co-localize around the lipid droplet and that the spatial distance between PEDF and ATGL was less than 20 nm (Figure 5C).

### PEDF and 44mer-induced TG degradation depend on the ATGL lipolysis activity

**Figure 6:**
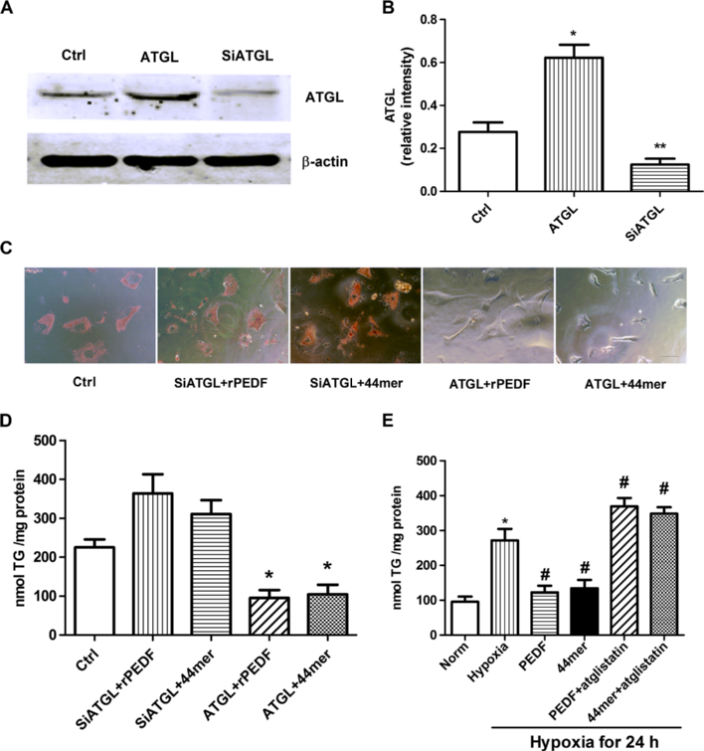
**PEDF and 44mer-induced TG degradation depend on the ATGL lipolysis activity** ***.*** **A**: western blots for ATGL of untreated control cell samples, samples treated with ATGL overexpression, and samples treated with ATGL siRNA compared with β-actin are shown. **B**: densitometry of the western blots shown in **(A)** normalized to β-actin. *n* = 3, values are means ± SD. *P < 0.05 compared with control samples. **P < 0.05 compared with control samples and ATGL group. **C**: oil red O staining of untreated control cardiomyocytes, cardiomyocytes treated with siATGL + rPEDF, cardiomyocytes treated with siATGL + 44mer, cardiomyocytes treated with ATGL + rPEDF and cardiomyocytes treated with ATGL + 44mer. Scale bar, 20 μm. **D**: quantification of TG content of cardiomyocytes samples shown in **(A)**, *P < 0.05 compared with control group. *n* = 3, Values are means ± SD. **E**: quantification of TG content in the presence of ATGL inhibitor atglistatin. *n* = 4, *P < 0.05 compared with control group; #*P* < 0.05 compared with hypoxia group. Values are means ± SD.

To test whether ATGL is required for PEDF or 44mer TG degradation, we used siRNA to knock down the endogenous ATGL expression in cardiomyocytes. As shown in Figure 6A, a band near 55 kDa was observed in the ATGL overexpression group, whereas the band was less pronounced in the siATGL group than in the control. rPEDF and 44mer administration into cardiomyocytes medium decreased the TG content when ATGL was overexpressed; however, these effects were abolished when ATGL was downregulated (Figure 6C and D). The necessity of the lipolysis activity of ATGL for PEDF and 44mer-mediated TG degradation was further confirmed by culturing cardiomyocytes with an ATGL-specific inhibitor, atglistatin, prior to the addition of PEDF and 44mer. Atglistatin attenuated the PEDF and 44mer-induced TG lipolysis activation of cardiomyocytes at 10 μmol/L. (Figure 6E). These results suggest that PEDF and 44mer-induced TG degradation depend on ATGL.

## Discussion

PEDF is a multifunctional, pleiotropic protein with antioxidant and antithrombotic properties. It is strongly expressed in the heart and linked to atherosclerosis [19]. Our group found that PEDF can improve functional recovery in an AMI rat model. However, the reason for this improvement was poorly understood. In this article, we demonstrated the importance of PEDF and its functional peptides 44mer in hypoxia/ischemia-induced lipid accumulation in cardiomyocytes, and we also demonstrated the importance of the PEDF-ATGL interaction in regulating the TG content of the rat heart. PEDF and 44mer may improve cardiac function after infarction via these mechanisms.

Under normal physiologic conditions, the heart utilizes fatty acids as its chief energy substrate. Because the capacity for TG storage in the cardiomyocyte is limited, the uptake and oxidation of fatty acids is tightly coupled [20]. Under adverse circumstances like myocardial ischemia, the heart switches the primary energy source to glucose instead of fatty acids to conserve oxygen during ATP generation [21]. If the supply of FFAs exceeds the capacity for FAO, the excess FFAs are incorporated into TG, which results in cardiac steatosis. Excess lipid accumulation in the heart has been shown to manifest as “cardiac lipotoxicity”, which leads to cardiac myocyte apoptosis and subsequent contractile dysfunction [22]. Bilheimer et al. showed that the most pronounced accumulation of labeled lipids after 6 to 24 h of LAD occlusion was located in the tissue surrounding the ischemic myocardium [23]. In this study, histological analyses of the infarcted hearts showed that lipid droplets continued to accumulate within cardiac myocytes 2, 4 and 6 weeks after myocardial ischemia. We also showed that the expression of PEDF dramatically decreased in biopsies from the left ventricle of ischemic rat hearts compared with biopsies from nonischemic left ventricles, which is consistent with the studies that PEDF is down-regulated in human cardiac myocytes and human cardiac fibroblasts by anoxia [24]. These findings indicate that PEDF may play an important role in preventing lipid accumulation in the ischemic heart. Therefore, in this study, we focused our attention on overexpressing PEDF in vivo and in vitro in order to investigate the effects and elucidate possible mechanisms.

PEDF is a multifunctional protein whose roles is divergent and cell type-specific. Early studies clearly demonstrated a pro-survival function in neuronal cells [25] whereas subsequent reports showed that PEDF can promote the apoptosis of activated endothelial cells [26]. As a secreted glycoprotein, the binding of PEDF to its receptor is the first step that mediates its biological effects. A 55-kDa phospholipase-linked membrane protein, the independent phospholipase A2 (PLA2)/adipose triglyceride lipase (ATGL), has been identified as a PEDF receptor [11]. ATGL is a specific TG hydrolase. It plays a critical role in the degradation of TG stored in intracellular lipid droplets [12,27] and possesses TG lipase and acylglycerol transacylase activities in adipose systems. In humans, mutations in the ATGL gene (PNPLA2) are associated with a rare inherited disorder annotated as neutral lipid storage disease with myopathy (NLSD-M) [28]. To date, most of published experimental work has focused on the role of ATGL in this process. In ATGL-ko mice, the excess accumulation of TGs in the heart causes cardiac abnormalities, and these mice suffer from premature death due to severe cardiac steatosis and dysfunction [29,30]. However, the exact role of PEDF in the process of TG degradation after myocardial infarction remains poorly understood. Our results showed that the TG content was higher in siPEDF rats than in PEDF-overexpressing rats after myocardial infarction. Both rPEDF and 44mer administration stimulate the TG degradation in cultured cardiomyocytes. Thus, our results indicated that PEDF and 44mer have an important effect on the degradation of TGs in cardiomyocytes and that the decreased expression of PEDF is essential for the accumulation of TGs in hypoxic cardiomyocytes and the ischemic myocardium in rats. However, PEDF and 44mer could not directly promote the metabolism of TGs. After decreasing the level of ATGL, the corresponding variations began to disappear. These results conclusively demonstrated that the PEDF and 44mer can stimulate ATGL activity.

Chung et al. showed that PEDF regulates the hepatocyte TG content by binding to ATGL on lipid droplets [31]. But, it was still uncertain whether PEDF and 44mer have directly affects on ATGL in cardiomyocytes. Certain proteins regulate the ATGL enzyme activity. CGI-58 (comparative gene expression protein-58, also known as hydrolase domain containing protein 5, ABHD5) functions as an activator protein of ATGL and exhibits no intrinsic TG hydrolase activity [32]. ATGL activity primarily increases under acute lipolytic stimulation via interaction with CGI-58. By detecting the expression levels of CGI-58 and ATGL, we found that PEDF and 44mer did not increase the expression of CGI-58 or ATGL. Moreover, we provided evidence in cardiomyocytes to suggest that PEDF and ATGL co-localized in a pattern consistent with lipid droplets. We also observed by immunoelectron microscopy that the spatial distance between PEDF and ATGL is less than 20 nm. This phenomenon indicated that the interaction between PEDF and ATGL was direct. Thus, we speculated that PEDF stimulates ATGL lipase activity by linking itself to ATGL.

## Conclusions

In summary and to the best of our knowledge, this study is the first to show that PEDF down-regulation contributes to the defect of ATGL transacylase activity, which subsequently leads to TG storage in cardiomyocytes in hypoxic conditions. PEDF and its functional peptides 44mer may offer a novel promising strategy for the treatment of myocardial TG accumulation after AMI. Yet, some studies indicated the increased ectopic lipid storage per se does not cause lipotoxicity [33]. But, the lipolysis of TGs to free FAs enables the production of ATP in the mitochondria via β-oxidation. PEDF may also exert survival actions in the cardiomyocytes via a lipidsignaling pathway mediated by ATGL. However, the detailed products of TG degradation remain unknown. Therefore, future investigations are needed to clarify the species of FFAs by which PEDF and 44mer protect cardiac function from ischemic injury.

## Abbreviations

PEDF: Pigment epithelium-derived factor
AMI: Acute myocardial infarction
LV: Lentivirus
ATGL: Adipose triglyceride lipase
LAD: Left-anterior descending coronary artery
TG: Triglyceride
CGI-58: Comparative gene expression protein-58
